# Prevalence of fatty liver disease and its associated factors among Jordanian patients with type 2 diabetes mellitus: A cross-sectional study

**DOI:** 10.1016/j.amsu.2021.102677

**Published:** 2021-08-06

**Authors:** Malik H. Almahmoud, Nahla M. Al Khawaja, Arwa Alkinani, Yousef Khader, Kamel M. Ajlouni

**Affiliations:** a The National Center for Diabetes, Endocrinology and Genetics, The University of Jordan, Amman, Jordan; b The National Center for Diabetes, Endocrinology and Genetics, Jordan; c Department of Public Health, Jordan University of Science and Technology (JUST), Irbid, Jordan

**Keywords:** Non-alcoholic fatty liver disease, Diabetes mellitus, Risk factors, Prevalence

## Abstract

**Background:**

Diabetes mellitus (DM) is a well-known risk factor for Non-alcoholic fatty liver disease (NAFLD). Patients with type 2 DM (T2DM) who have NAFLD are at a higher risk of developing advanced stages of liver disease, including fibrosis, cirrhosis, and hepatocellular carcinoma compared to non-diabetic patients. This study aimed to estimate the prevalence of NAFLD among patients with T2DM, using hepatic ultrasonographic changes combined with derangement of hepatic transaminases level.

**Materials and methods:**

This cross-sectional study was conducted at the National Center for Diabetes, Endocrinology and Genetics (NCDEG) in Amman, Jordan. A total of 408 patients with T2DM and 90 non-diabetic subjects were included in this study. Body mass index (BMI), waist circumference, glycosylated hemoglobin (HbA1c), lipid parameters and abdominal ultrasonography were measured.

**Results:**

Using the ultrasonographic criteria for the diagnosis of NAFLD, the prevalence of NAFLD was 80.4 % and 53.3 % among diabetic and non-diabetic participants, respectively. Among the diabetic participants, 25 %, 40.4 %, and 15 % had mild, moderate, and severe grades of steatosis, respectively. On the other hand, 24.4 %, 21.1 %, and 7.8 % of the non-diabetic participants had mild, moderate, and severe grades of steatosis, respectively. Diabetic patients between 25 and 45 years of age, patients with overweight or obesity, patients with increased waist circumference were significantly at higher risk of having NAFLD. High TG, lower HDL, elevated AST and ALT, and using sulfonylureas and metformin versus using metformin only were significantly associated with increased odds of having NAFLD.

**Conclusions:**

NAFLD is highly prevalent among patients with T2DM. Overweight or obesity, abnormal cholesterol levels and treatment with sulfonylureas were significantly associated with NAFLD.

## Highlights

• NAFLD is a highly prevalent condition among patients with T2DM in Jordan.
• Overweight and obesity are associated with increased risk of NAFLD.
• Weight reduction and lifestyle modification are recommended strategies to prevent NAFLD.

## 1 Introduction

Non-alcoholic fatty liver disease (NAFLD) is one of the most highly prevalent chronic liver disorders worldwide, irrespective of age, gender and ethnicity [1]. NAFLD is characterized by the deposition of fat in the liver, leading to a spectrum of disorders including simple steatosis, steatohepatitis, cirrhosis and hepatocellular carcinoma (HCC) in the absence of excessive alcohol intake [2].

The prevalence of NAFLD is variable depending on the diagnostic criteria used to define NAFLD in the general population. The prevalence is almost 15–20 % based on derangement of hepatic transaminase levels and reaches to 20–46 % when hepatic ultrasonographic changes were used to define NAFLD [3,4].

Diabetes mellitus (DM) is a well-known risk factor for NAFLD, in addition to obesity, hyperlipidemia, and metabolic syndrome [1]. The prevalence of NAFLD in patients with type 2 diabetes mellitus (T2DM) worldwide was estimated at 34–94 % [5]. This common association could be explained by the defective lipid metabolism with triglyceride deposition in the liver, as a result of insulin resistance [6]. Patients with T2DM who have NAFLD are at a higher risk of developing advanced stages of liver disease, including fibrosis, cirrhosis, and hepatocellular carcinoma, in comparison to non-diabetic patients [1,7].

A previous study in Jordan reported that the prevalence of fatty liver in T2DM using derangement in hepatic transaminases ALT, AST, and combined ALT and AST was 10.4%, 5.4 %, and 4.5 % respectively [8]. This study aimed to estimate the prevalence of fatty liver among patients with T2DM, using hepatic ultrasonographic changes combined with derangement of hepatic transaminases level.

## 2 Methods

### 2.1 Study design and settings

A cross-sectional study was conducted among adult patients with T2DM (≥18 years old) who attended National Center for Diabetes, Endocrinology, and Genetics (NCDEG) clinics in Amman, Jordan, between November 2019 and February 2020. Patients with a known history of liver disease, pregnant women, patients who reported alcohol consumption, and those who were on amiodarone, anti-epileptic drugs, or methotrexate were excluded from the study. A systematic sample of every fifth diabetic patient meeting the inclusion criteria (Non-pregnant adult aged ≥ 18 years with T2DM) and the exclusion criteria was selected. The non-diabetic participants were selected randomly from the patients’ relatives accompanying them, who were willing to volunteer in the current study. They were not known to have diabetes mellitus nor liver disease and not on medications that are well known to have hepatotoxicity. They were subjected for laboratory tests to confirm normal fasting blood glucose and Glycosylated hemoglobin (HbA1c) levels. If the patient was not fasting, they requested to come in the next day to have their fasting blood glucose measured.

The study was registered in the Research Registry (www.researchregistry.com) with a unique identifying number “researchregistry6936”. The manuscript has been reported in line with the STROCSS criteria [9].

### 2.2 Data collection

Patients were interviewed face-to-face using a structured questionnaire to collect information about sociodemographic characteristics. Laboratory data were extracted from the medical files. Anthropometric measurements, including height, weight, and waist circumference were measured while the subject was in light clothes and barefooted. Waist circumference was measured at the end of a normal expiratory phase using a tape measure around the abdomen at the level of the iliac crest [10]. According to the anthropometric cutoff values in Jordanian adults, a waist circumference of 88.5–91.8 cm in men and from 84.5 to 88.5 cm in women was considered normal [11]. Height measurement was taken using a Harpenden stadiometer to the nearest 0.5 cm, while the patient's back was in a vertical line to the stadiometer and parallel to the shoulder blades. Weight was measured to the nearest 0.5 kg using a Seca weight scale. Waist to height ratio was considered normal if waist to height ratio was ≤0.5, and elevated if it was> 0.5 [11]. Body mass index (BMI) was calculated as weight in kilogram (kg) divided by the height squared (m^2^). The World Health Organization was used to classify BMI [12]. BMI standard for Caucasian was used because Jordanians are classified as Caucasians.

### 2.3 Laboratory measurements

Venous blood samples were collected in the morning from patients after fasting for at least 8 h and tested for glucose, total cholesterol (TC), triglycerides (TG), low-density lipoprotein (LDL) and high-density lipoprotein (HDL) using the method of Enzymatic Colorimetric, COBAS INTEGRA, supplied by Roche Diagnostics. Glycosylated hemoglobin (HbA1c) was measured using the method of High-Performance Liquid Chromatography, Bio-Rad. Aspartate aminotransferase (AST) and Alanine aminotransferase (ALT) were measured by the quantitative determination of the catalytic enzyme activity, COBAS INTEGRA systems, supplied by Roche Diagnostics. Elevated AST and ALT levels were defined as > 40 U/L for males and >32 U/L for females, and >41 U/L for males and >33 U/L for females, respectively, according to the center's laboratory assay. Serological tests for the hepatitis B surface antigen (HBsAg), hepatitis B surface antibody (HBsAb), and hepatitis C virus (HCV) antibodies were assessed for the study participants.

### 2.4 Radiographs

Patients were then referred to the radiological department for a hepatic ultrasonography examination by a registered radiologist at NCDEG. The examination was performed using a sensitive ultrasound machine with a 3–5 MHZ convex transducer. Longitudinal and transverse scanning was performed and the texture with measurement of the liver span was recorded. The normal liver parenchyma has a homogenous echotexture, with echogenicity equal or slightly more than that of the spleen and renal cortex. The fatty liver showed higher echogenicity than the renal cortex on both sides. Different grading of steatosis has been classified based on the intensity of the echogenicity. When the echogenicity was increased, this was considered to be grade 1 steatosis (mild). When the branches of the portal vein were obscured it was considered to be grade 2 steatosis (moderate), and when the echogenicity of the liver covered the diaphragmatic outline, it was classified as grade 3 steatosis (severe) [13].

### 2.5 Ethical considerations

This study was approved by the Institutional Review Board at NCDEG (1/2019). Confidentiality has been assured to patients. Informed consent was signed by all participants. The study is registered in the Research Registry (researchregistry6936).

### 2.6 Statistical analysis

The Statistical Package for Social Sciences (SPSS) version 21 was used for data analysis. Mean and standard deviation was used to describe continuous variables and percentages for categorical variables. The association of fatty liver with different variables was determined by Chi-square (χ2) test. Multivariate logistic regression was used to determine the factors associated with the presence of fatty liver. A P-value of <0.05 was considered statistically significant.

## 3 Results

This study included 408 diabetic patients (218 females and 190 males) and 90 non-diabetic individuals as a control group (60 females and 30 males). The mean age of the study participants was 56.3 ± 10.4 years for the diabetic patients and 52.3 ± 14.2 years for the control group. There were statistical differences between the diabetic and non-diabetic participants, in terms of age, BMI, waist circumference, and waist to height ratio (Table 1).

**Table 1:** Socio-demographic and clinical characteristics of the study participants.

Variables	Diabetics (n = 408) N (%)	Non-diabetics (n = 90) N (%)	P-VALUE
**Gender**			0.054
Male	190 (46.6)	30 (33.3)	
Female	218 (53.4)	60 (66.7)	
**Age (years)**	56.3 ± 10.4	52.3 ± 14.2	0.002
25–45	60 (14.7)	28 (31.1)	0.001
46–65	268 (65.7)	44 (48.9)	
>65	80 (19.6)	18 (20.0)	
**BMI (kg/m2)**	32.63 ± 5.8	29.7 ± 6.3	0.000
18.5–24.9	29 (7.1)	35 (38.9)	0.000
25–29.9	110 (27.0)	18 (20.0)	
≥30	269 (65.9)	37 (41.1)	
Waist circumference (cm)*	102.9 ± 12.1	93.2 ± 12.9	0.000
Normal	56 (13.7)	39 (43.3)	0.000
elevated	352 (86.3)	51 (56.7)	
**Waist/height ratio**			
Normal (<0.5)	12 (2.9)	23 (25.6)	
Abnormal (≥0.5)	396 (97.1)	67 (74.4)	0.000
**Lipids (mg/dL)**			
LDL > 100	235 (57.6)	52 (58.4)	0.886
TG > 150	220 (53.9)	39 (43.3)	0.069
HDL < 40 (males)	97 (51.1)	9 (45.0)	0.607
HDL < 50 (females)	140 (64.2)	19 (47.5)	0.046
**FATTY LIVER**			
Yes	328 (80.4)	48 (53.3)	
No	80 (19.6)	42 (46.7)	0.000
**Liver enzymes (U/L)**			
ALT (ELEVATED)	104 (25.5)	18 (20.0)	0.273
AST (ELEVATED)	62 (15.2)	11 (12.2)	0.470
ALT and AST (ELEVATED)	51 (12.5)	8 (8.9)	0.337
**Steatosis grade**			
Grade 0 (no fatty liver)	80 (19.6)	42 (46.7)	
Grade 1 (mild)	102 (25.0)	22 (24.4)	
Grade 2 (moderate)	165 (40.4)	19 (21.1)	
Grade 3 (severe)	61 (15.0)	7 (7.8)	0.000

Using the ultrasonographic criteria for the diagnosis of NAFLD, the prevalence of NAFLD was 80.4 % and 53.3 % among diabetic and non-diabetic participants, respectively. Among the diabetic participants, 25 %, 40.4 %, and 15 % had mild, moderate, and severe grades of steatosis, respectively. On the other hand, 24.4 %, 21.1 %, and 7.8 % of the non-diabetic participants had mild, moderate, and severe grades of steatosis, respectively.

Among diabetic participants, patients with NAFLD were significantly younger than patients without NAFLD. Furthermore, they had higher BMI, higher waist circumference, elevated TG level, and lower HDL level. As shown in Table 2, 30.2 % and 18.3 % of patients with NAFLD had elevated ALT and AST levels, respectively.

**Table 2:** Clinical characteristics of T2DM patients with and without NAFLD.

Variables	NAFLD (n = 328), No. (%)	No NAFLD (n = 80), No. (%)	P-value
**Gender**			
Male	148 (45.1)	42 (52.5)	
Female	180 (54.9)	38 (47.5)	0.236
**Age (years)**			
25–45	54 (16.5)	6 (7.5)	
46–65	222 (67.7)	46 (57.5)	
>65	52 (15.9)	28 (35)	0.000
**BMI (kg/m2)**			
18.5–24.9	15 (4.6)	14 (17.5)	
25–29.9	82 (25)	28 (35)	
≥30	231 (70.4)	38 (47.5)	0.000
**Waist circumference (cm)**			
Normal	33 (10.1)	23 (28.7)	
elevated	295 (89.9)	57 (71.2)	0.000
**Waist/height ratio**			
Normal (<0.5)	4 (1.2)	8 (10)	
Abnormal (≥0.5)	324 (98.8)	72 (90)	0.000
**Diabetes duration (years)**			
<5	119 (36.3)	28 (35)	
5–10	119 (36.3)	26 (32.5)	
11–15	57 (17.4)	15 (18.8)	
>15	33 (10.1)	11 (13.8)	0.761
**Lipids (mg/dL)**			
LDL > 100	188 (57.3)	47 (58.8)	0.816
TG > 150	191 (58.2)	29 (36.2)	0.000
HDL < 40 (males)	84 (56.8)	13 (31)	0.003
HDL < 50 (females)	126 (70)	14 (36.8)	0.000
**Liver enzymes (U/L)**			
ALT (ELEVATED)	99 (30.2)	5 (6.2)	0.000
AST (ELEVATED)	60 (18.3)	2 (2.5)	0.000
ALT and AST (ELEVATED)	50 (15.2)	1 (1.2)	0.001
**Antidiabetic agents**			
Metformin	109 (33.2)	33 (41.2)	
Metformin + Insulin	86 (26.2)	25 (31.2)	
Metformin + Insulin + Sulfonylurea	41 (12.5)	9 (11.2)	
Metformin + Sulfonylurea	92 (28.0)	13 (16.2)	0.148

Biochemical and physical characteristics of the study participants, according to the steatosis grade Are shown in Table 3. The mean age of patients with severe steatosis was 55.2 ± 9.2 years in comparison to 59.6 ± 11.7 years in diabetic patients with no radiological features of fatty liver (P-value 0.003).

**Table 3:** Clinical and biochemical characteristics of diabetic participants according to steatosis grade.

Variables	No fatty liver No. (%)	Mild steatosis No. (%)	Moderate steatosis No. (%)	Severe steatosis No. (%)	P-value
**Age (years)**	59.6 ± 11.7	57.3 ± 9.6	54.5 ± 10.4	55.2 ± 9.2	0.003
**Gender**					
Male	42 (52.5)	50 (49)	84 (50.9)	14 (23)	
Female	38 (47.5)	52 (51)	81 (49.1)	47 (77)	0.001
BMI (kg/m^2^)	29.4 ± 4.9	32.1 ± 4.9	33.4 ± 5.6	35.7 ± 7.1	0.000
Waist circumference (cm)	96.7 ± 11.3	101.8 ± 10.8	105.5 ± 11.7	106.0 ± 13.1	0.000
Duration of diabetes (years)	9.1 ± 7.3	9.1 ± 7.1	8.1 ± 6.1	7.2 ± 4.7	0.212
HbA1c (%)	7.4 ± 1.5	7.6 ± 1.9	7.9 ± 1.6	7.8 ± 1.5	0.166
Total cholesterol (mg/dL)	162.3 ± 42.1	175.5 ± 49.1	166.5 ± 49.4	167.4 ± 48.2	0.282
HDL (mg/dL)	49.6 ± 13.5	45.4 ± 12.8	42.0 ± 11.5	41.2 ± 10.1	0.000
LDL (mg/dL)	108.9 ± 35.9	115.5 ± 39.2	114.2 ± 38.0	106.7 ± 37.7	0.381
TG (mg/dL)	146.9 ± 78.8	162.7 ± 77.5	202.9 ± 115.4	196.4 ± 92.4	0.000
ALT (elevated)	5 (6.2)	17 (16.7)	50 (30.3)	32 (52.5)	0.000
AST (elevated)	2 (2.5)	8 (7.8)	28 (17)	24 (39.3)	0.000

Diabetic patients with steatosis have significantly higher BMI and waist circumference in comparison to those without a fatty liver. Furthermore, diabetic patients with steatosis had lower HDL and high TG levels, compared to those without a fatty liver. With the advancement of the stages of fatty liver, the hepatic transaminases tend to be elevated as a sequel of the degree of the hepatic injury caused by the fat accumulation. No statistical differences were found among patients with different degrees of steatosis in terms of duration of diabetes, HbA1c level, total cholesterol, and LDL level.

Table 4 shows the multivariate analysis of factors associated with fatty liver among diabetic patients. The likelihood of fatty liver was significantly higher in diabetic patients between 25 and 45 years of age, in comparison to those older than 65 years of age. Overweight diabetic patients had a 2.72 times higher risk of developing NAFLD than patients with normal BMI. Similarly, obese diabetic patients had a 4.77 times higher risk of developing NAFLD compared to normal BMI patients. Elevated waist circumference (OR = 3.05) was associated with an increased risk of having NAFLD. Patients with high TG and lower HDL cholesterol levels were 1.91 times and 2.72 times more likely to have NAFLD, respectively. Patients with elevated AST and ALT were 4.87 times and 6.36 times more likely to have NAFLD, respectively. Diabetic patients treated with sulfonylureas and metformin were 2.34 times more at risk of having NAFLD in comparison to patients using metformin only.

**Table 4:** Multivariate analysis of factors associated with fatty liver among diabetic patients.

Variable	Odds ratio (95 % CI)	P value
Age (years)		
25–45	1	
46–65	0.56 (0.21–1.51)	0.251
>65	0.25 (0.09–0.72)	0.010
BMI (kg/m2)		
18.5–24.9	1	
25–29.9	2.72 (1.06–6.95)	0.037
≥30	4.77 (1.95–11.65)	0.001
Increased waist circumference (cm)	3.40 (1.80–6.44)	0.000
Elevated ALT	4.87 (1.86–12.77)	0.001
Elevated AST	6.37 (1.48–27.32)	0.013
Elevated TG	1.91 (1.21–3.02)	0.006
Abnormal HDL	2.72 (1.58–4.71)	0.000
Antidiabetic agents		
Metformin	1	
Metformin + Insulin	0.80 (0.41–1.56)	0.512
Metformin + Insulin + Sulfonylurea	0.97 (0.39–2.41)	0.948
Metformin + Sulfonylurea	2.34 (1.08–5.09)	0.032

## 4 Discussion

NAFLD is highly prevalent among Jordanians with type 2 diabetes mellitus. The prevalence of NAFLD was found to be 80.4 % in the current study. The prevalence of elevated ALT and AST was 25.5 % and 15.2 %, respectively. In comparison to a previous study that was conducted in the same center about 10 years ago, the prevalence of elevated ALT and AST were 10.4 % and 5.4 %, respectively [8]. This difference could be related to the baseline characteristics of the study population; two-thirds of our study population had BMI ≥30 kg/m^2^ compared to 56.4 % of the previous study population.

The prevalence of NAFLD in the current study is comparable to a previously reported prevalence from a meta-analysis that ranged between 29.6% and 87 % [14]. This high prevalence is related to the significant association of multiple metabolic risk factors, such as insulin resistance, obesity, and dyslipidemia between type 2 diabetes mellitus and NAFLD. Furthermore, other studies have considered NAFLD as a hepatic presentation of metabolic syndrome [15].

In contrast to previously reported studies [[16], [17], [18], [19]], gender distribution in the prevalence of NAFLD was not significant. Population-based studies have previously reported the association of NAFLD with the male gender, and this association is related to the protective role of female hormones and lower lipid levels among females [16]. However, the association with gender in our study could be explained by the decline of the impact of female hormones on the prevalence of NAFLD by the higher prevalence of overweight and obesity in female patients in comparison to male patients with NAFLD (P-value = 0.023). Also, 70 % of female patients had an abnormal HDL level compared to 56.8 % of male patients (P-value = 0.013). This significantly higher prevalence of low HDL levels among our female patients could also lessen the effect of the protective female hormone on the prevalence of NAFLD.

Our study has shown that obesity is a significant predictor of NAFLD, and the higher the BMI the higher the likelihood of NAFLD among patients with T2DM. Similar to our findings, several studies have reported the significant association between obesity and NAFLD [[20], [21], [22]]. This association is explained by insulin resistance as a common pathophysiological mechanism involved in the pathogenesis of obesity and NAFLD [23].

Similar to several previously reported studies, elevated waist circumference and waist/hip ratio are significant predictors of NAFLD among diabetic patients [17,24,25]. The fat distribution also plays an important role in the development of NAFLD, visceral obesity measured by waist circumference and waist/hip ratio has a hepatotoxic effect through a release of several inflammatory markers, such as tumor necrosis factor (TNF) and interleukin-6 (IL-6), in addition to fatty acid accumulation in the liver parenchyma [26]. Our data emphasize the importance of weight loss in the prevention of NAFLD and the improvement of the histological scoring of hepatic steatosis in this disorder as previously reported [27].

The prevalence of NAFLD was 16.5 %, 67.7 %, and 15.9 % in the patient aged 25–45 years, 46–65 years, and ˃65 years, respectively. Hence, the older diabetic patients were less likely to have NAFLD than younger patients (OR: 0.25, 95%CI: 0.08–0.72, p = 0.010). Similar to our findings, other studies have shown that the prevalence of NAFLD decreases with age [28,29]. Although risk factors for NAFLD such as obesity, dyslipidemia, and metabolic syndrome increase with age, it seems that aging is a protective factor for NAFLD. Nevertheless, aging carries a higher risk of advanced liver fibrosis and cirrhosis in NAFLD patients [30].

In our study, we have seen that NAFLD is positively associated with low HDL levels (OR: 2.73, 95%CI: 1.57–4.71, p = 0.000) and elevated TG levels (OR: 1.91, 95%CI: 1.21–3.02, P = 0.006). The same results were observed in previous studies [19]. In general, hypertriglyceridemia and increased TG/HDL ratio are involved in the pathogenesis of NAFLD through the accumulation of fat in the liver parenchyma [23].

Among antidiabetic medications, sulfonylurea was found to be linked to increased risk for NAFLD (OR: 2.34, 95%CI: 1.1–5.1, P = 0.032). However, in our study insulin was not associated with an increased risk of NAFLD in contrary to other studies [15,31]. Nevertheless, Tang An et al. have shown that insulin glargine is associated with hepatic fat reduction [32].

One of the current study limitations is using ultrasound for the diagnosis of fatty liver. It is known that ultrasound has a sensitivity of 82–94 % and specificity of (60–95 %) for the diagnosis of NAFLD [28]. While, the gold standard test for the diagnosis of NAFLD is a liver biopsy [25]. Furthermore, we have not studied the doses of different antidiabetic medications that could influence the prevalence of the fatty liver.

On the other hand, this study was conducted in a diabetic clinic, which will evaluate the real prevalence of NAFLD among diabetic patients; while enrollment of patients from the hepatology clinic will mostly include symptomatic patients and/or patients with abnormal plasma transaminases, or advanced stages of liver disease.

In conclusion, NAFLD is highly prevalent in T2DM patients in Jordan. Obesity, increased waist circumference, low HDL level, elevated TG level and sulfonylureas are associated with an increased risk for developing NAFLD. Attention should be paid to minimize the burden of NAFLD among diabetic patients. Weight reduction, with lifestyle modification and health education for T2DM patients, are recommended strategies that play a crucial role in the prevention of NAFLD. Early treatment of abnormal lipid parameters in the form of controlling HDL and TG levels is important to prevent NAFLD and its negative impact on the patient's life.

## Ethical approval

This study was approved by the Institutional Review Board at NCDEG (1/2019). Confidentiality has been assured to patients.

## Funding

No funding

## Author statement

Malik H. Almahmoud: study concept/design, data collection, data interpretation, writing the paper.

Nahla M. Al Khawaja: study concept/design, data collection, writing the paper.

Arwa Alkinani: study concept/design, data collection, data interpretation.

Yousef Khader: study concept/design, data collection, data interpretation, data analysis, paper revision.

Kamel M Ajlouni: study concept/design, data collection, data interpretation, paper revision.

## Registration of research studies

1 Name of the registry:
2 Unique Identifying number or registration ID:
3 Hyperlink to your specific registration (must be publicly accessible and will be checked):

## Guarantor

Malik H. Almahmoud.

## Provenance and peer review

Not commissioned, externally peer-reviewed.

## Consent

Informed consent was signed by all participants.

## Declaration of competing interest

No conflict of interest.
